# Interaction of Ceramic Implant Materials with Immune System

**DOI:** 10.3390/ijms24044200

**Published:** 2023-02-20

**Authors:** Guzel Rafikova, Svetlana Piatnitskaia, Elena Shapovalova, Svyatoslav Chugunov, Victor Kireev, Daria Ialiukhova, Azat Bilyalov, Valentin Pavlov, Julia Kzhyshkowska

**Affiliations:** 1 Laboratory of Immunology, Institute of Urology and Clinical Oncology, Bashkir State Medical University, 450008 Ufa, Russia; 2 Institute of Fundamental Medicine, Bashkir State Medical University, 450008 Ufa, Russia; 3 Department of Chemistry, Tomsk State University, 634050 Tomsk, Russia; 4 Skolkovo Institute of Science and Technology, 121205 Moscow, Russia; 5 Department of Applied Physics, Ufa University of Science and Technology, 450076 Ufa, Russia; 6 Bashkir State Medical University, 450008 Ufa, Russia; 7 Institute of Transfusion Medicine and Immunology, Mannheim Institute of Innate Immunosciecnes (MI3), Medical Faculty Mannheim, Heidelberg University, 69117 Mannheim, Germany; 8 German Red Cross Blood Service Baden-Württemberg, 68167 Mannheim, Germany

**Keywords:** bioceramic, innate immunity, macrophage, inflammation, healing, regenerative medicine, immune response

## Abstract

The immuno-compatibility of implant materials is a key issue for both initial and long-term implant integration. Ceramic implants have several advantages that make them highly promising for long-term medical solutions. These beneficial characteristics include such things as the material availability, possibility to manufacture various shapes and surface structures, osteo-inductivity and osteo-conductivity, low level of corrosion and general biocompatibility. The immuno-compatibility of an implant essentially depends on the interaction with local resident immune cells and, first of all, macrophages. However, in the case of ceramics, these interactions are insufficiently understood and require intensive experimental examinations. Our review summarizes the state of the art in variants of ceramic implants: mechanical properties, different chemical modifications of the basic material, surface structures and modifications, implant shapes and porosity. We collected the available information about the interaction of ceramics with the immune system and highlighted the studies that reported ceramic-specific local or systemic effects on the immune system. We disclosed the gaps in knowledge and outlined the perspectives for the identification to ceramic-specific interactions with the immune system using advanced quantitative technologies. We discussed the approaches for ceramic implant modification and pointed out the need for data integration using mathematic modelling of the multiple ceramic implant characteristics and their contribution for long-term implant bio- and immuno-compatibility.

## 1. Introduction

The use of medical implants provides advanced solutions for major fields of clinical medicine, such as oncology, cardiovascular medicine, orthopedics and dentistry. For example, the global market for knee implants in 2021 is estimated to reach USD 9.8 billion [1], and the global dental implant market in 2021 accounted for USD 3.9 billion [2].

Implanted materials are commonly recognized by the immune system as foreign bodies, and their installation may be accompanied by the development of inflammatory reactions that can lead to implant rejection [3]. Macrophages are key elements of the innate immune system that recognize the shape, surface structure and material of the implants, and orchestrate the reactions of the tissues at all the stages of implant integration, starting with initial acute inflammatory reaction followed by resolution of inflammation and healing, fibrotic cap formation and long-term integration [3,4,5]. Accumulated data provide evidence for the specific transcriptional and secretory profiles induced in the innate immune cells by distinct implant materials, justifying large experimental effort for researchers to develop new, improved biomaterials with the most compatible composition.

Each type of material can cause different biological complications when implanted in vivo. An adverse immune reaction can be the cause of prosthesis failure on a mechanical level [4]. For example, metals exhibit corrosive behavior and cause metallosis in the peri-implant tissues, and non-biodegradable materials are prone to aseptic loosening. Aluminum-oxide-based ceramic implants stimulate a gradual development of a non-adhesive fibrous membrane at the implant’s surface, which reduces the strength of connection between these membranes and surrounding tissues. In the case of zirconium-based ceramics, hydroxyapatite (HA) and beta-phase tricalcium phosphate (β-TCP) ceramics, bone resorption around the implant is common. It results in the loss of natural bone tissue [6,7]. Ankylosis and decreased fracture resistance of the implanted materials are the typical outcomes of the use of bioglass materials [8].

The outlined processes are frequently explained by the mechanical or chemical properties of the materials themselves, while the impact of the biomedical response on the material microstructure is insufficiently considered.

The identification of the cellular and molecular interactions that mediate biological and immune responses to the foreign material is a pre-requisite for the advanced design of implant biomaterials with controlled integration at tissue-specific or organ-specific sites.

One of the main trends in current biomedical research is the development and production of materials for regenerative medicine based on bioceramics. They play an important role in tissue regeneration and repair because of the effect on cell proliferation and differentiation in the implantation area [3,4]. Ceramic materials based on calcium phosphate have the properties of osteoconductivity and biocompatibility due to their chemical similarity to the mineral phase of the native bone, but have worse mechanical properties in comparison with the materials traditionally used for implants [5].

Moreover, the interaction of osteogenic cells with ceramics leads to good bone integration and regeneration [3].

In order to provide a deeper understanding of the topic further discussed in this article, it is important to introduce some of the terms that will be frequently mentioned later in the text. Osteoconduction is the ability of bone-forming cells in the transplant area to move through the scaffold and slowly replace it with new bone over time. Another important property necessary for implants is osteoinduction, which can be defined as the process by which osteogenesis is induced [9,10].

Currently, intensive investigations are focused on the further enhancement of osteoconductivity and improvement in their mechanical properties. Further, the use of ceramic implants for the regeneration of soft tissue injuries is an emerging area of science. The development of such implants is still mostly at the investigative level in experimental models; however, the Food and Drug Administration (FDA) has cleared the implants for use in veterinary medicine, and FDA approval is pending for clinical use in daily practice in humans [11,12,13,14,15,16,17,18,19].

Despite all the advantages of bioceramics, their application today is still limited by the mechanical properties of the materials. The design and structures of implants most favorable for biointegration have reduced compressive, tensile strength and other mechanical properties compared to metal implants or natural bone tissues. Therefore, therapeutic manipulations with biological responses are strongly suggested in order to avoid further reductions in mechanical stability of the ceramic constructions. The local immune system at the site of implant installation has the ability not only to destroy the implant material itself by changing chemical milieu (ion concentrations, pH, oxidative reactions due to ROS production), but can also destroy the extracellular matrix structures, making implant fixation in the tissue inefficient or even impossible [20,21]

This review summarizes the beneficial and limiting properties of ceramic implants, highlights the features of ceramic materials, their interaction with different types of cells and tissues and also demonstrates the available knowledge and the gaps in knowledge about the interaction of ceramic implants with the immune system. In this article, we also identified areas for improvement and development of new ceramic materials for wider application and long-term integration, in particular by the optimization of their interaction with innate immunity.

## 2. Types of Ceramic Materials for Implant

Bioceramics are a large class of specially developed ceramics used to repair and reconstruct damaged structures [22,23]. Since ceramic materials are highly biocompatible and have a similar chemical composition to bone tissue, are bioactive, osteoconductive, osteoinductive and have good mechanical properties, ceramic implants are increasingly used for bone defect replacement [24,25,26,27,28,29,30].

Although bioceramics are biocompatible and rarely cause implant rejection, the available experimental data are not unequivocal. In a cohort of 108 patients, ossiculoplasties with bioceramic implants were performed, and patients were followed up clinically for a minimum of 7 years [31]. The rejection rate after 9–12 years was 21% (23 out of 108 patients). Histological integration rate was 79% (85 out of 108 patients), similar to results reported in the literature for both bioceramic and titanium implants [31].

All ceramic materials can be divided into two groups: biodegradable and non-biodegradable (see Table 1). Non-degradable ceramics include such materials as aluminum, titanium and zirconium ceramics; this type of material is not subject to degradation in vivo and has an insignificant material wear rate. These materials are used in orthopedics for bone injuries that cannot heal on their own, in the treatment of bone cancer to replace amputated skeletal parts and in dentistry for various dental issues [26,27,29,32,33,34,35,36]. However, non-degradable ceramics are not osteointegrative, which increases the risks of implant replacement.

The other type of material includes HA and β-TCP ceramics. The most important feature of this type is gradual degradation with gradual replacement by the patient’s own bone [33,50,51,52]. This behavior is ensured by their ability to stimulate osteoinduction and osteoconduction. This feature of the material allows its use in a wide range of medical applications: in orthopedics, in bone injuries, in dentistry and in oncology for bone cancer. Its biodegradability is beneficial to develop self-destructive drug delivery systems for treating cancer, osteomyelitis and periprosthetic infections [53,54,55,56,57,58,59,60,61].

Bioglass stands out among biodegradable materials due to its bioconductive and bioinductive properties [62,63,64,65]. Bioglass can be used for the replacement not only of bone defects but also soft tissues—skin implants, respiratory implants, cardiovascular implants, neurological implants for skin necrosis, myocardial necrosis, chronic obstructive pulmonary disease, peripheral nerve injuries, gastric ulcers and others [29,53]. The main limitations of ceramic materials are fragility and inconsistency of the mechanical properties with the native bone [3] (table in Section 3).

The broad possibilities and benefits of using biodegradable materials are being studied by numerous research groups. In cancer patients, the use of the implant construction enables the controlled and continuous release of the drug into the tumor microenvironment, which, in hand, leads to a reduction in the effects of systemic drug treatment [66,67]. Applications of nanohydroxyapatite/collagen scaffolds filled with doxorubicin, encapsulated in microspheres of poly (lactic-co-glycolic acid) (PLGA) in vitro demonstrated a prolonged drug release for a period of up to 28 days and significant growth inhibition of human osteosarcoma lineage cells (MG-63) [67]. In vivo experiments evaluated the immune response elicited by the subcutaneous implant on rats of these scaffolds, confirming their biocompatibility. The implants did not cause a significant inflammatory response, as assessed by histological evaluation of inflammation. The study did not search for local or systemic inflammatory markers [67]. The scaffold-treated group containing doxorubicin microspheres showed less tumor progression and fewer adverse effects than the control group or doxorubicin intraperitoneal injection group, demonstrating its promising anti-tumor effect [67]. However, the effects of such doxorubicin microspheres on intertumoral immunity, in particular, on tumor-associated macrophages, that can interfere with doxorubicin cytostatic effect on cancer cells [68], were not assessed in this study [68]

In addition to chemical drug delivery, bioceramics allow for the delivery of other biologically active substances. Human amniotic epithelial cells (hAECs) exhibit a strong capability to restore ovarian function in chemotherapy-induced premature ovarian failure. The injection of sodium alginate-bioglass (SA-BG)–hAECs composite hydrogel has been shown to be an effective strategy for ovarian tissue regeneration in mice. The injection restored follicle development, granulosa cell function and enhanced ovarian angiogenesis in mice’s ovarian system [69]. However, further studies using control groups comparing the delivery of hAECs to the ovaries by different pathways are needed. Further, the dynamic effects of alginate-bioglass (SA-BG)–hAECs composite hydrogel on the local immune system must be studied in detail, at least during the first 4 weeks after application.

**Figure 1:**
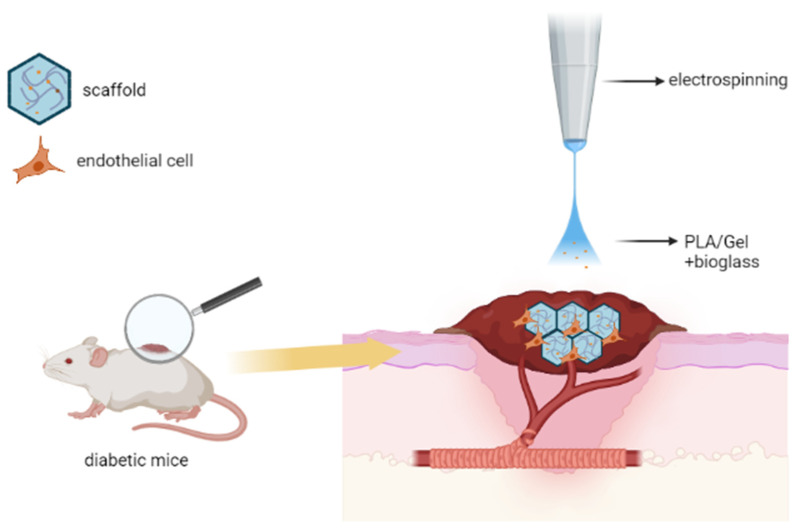
Application of micropatterned nanofibrous scaffold with bioglass nanoparticles for soft tissue regeneration (concept of this figure was developed in reference [70]).

Micropatterned nanofibrous scaffolds with bioglass nanoparticles encapsulated inside coaxial fibers were prepared by electrospinning and tested in a wound-healing model in diabetic mice [70]. The full-thickness 8 mm wounds were treated with different scaffolds. Healing of a wound in treated and non-treated mice was monitored on days 3, 5, 7, 9 and 14. The bioglass scaffold loaded with PLA/Gel coaxial fibers significantly enhanced wound healing to non-treated, PLA-treated or PLA/Gel coaxial-treated groups (Figure 1). Histological analysis showed that bioglass implants stimulated the formation of continuous epithelial tissue, essential for healing [70]. The study included an immunohistochemical search for vascular endothelial cells, but no search for markers of local or systemic inflammation.

Bioglass nanoparticles integrated in hydrogel with sodium alginate (BG-SA) were injected into the peri-infarct area of the rat myocardium [14]. After 4 weeks, a comparative analysis of the myocardium of rats receiving an injection of BG-SA or sodium alginate or normal saline was performed. The echocardiography showed a statistically significant improvement in the left ventricular ejection fraction in (ΔLVEF), specifically in the BG-SA group. Injection of BG-SA resulted in a statistically significant decrease in the infarct expansion index, increased number of capillaries accompanied by elevated VEGF levels and suppressed cardiomyocyte apoptosis [14]. The study did not look for markers of local or systemic inflammation.

Four consecutive cases of chronic osteomyelitis treated with antibiotic therapy, one-stage surgical debridement and bioglass implantation, were prospectively followed for a minimum of three years [71]. All patients achieved proper healing at the latest follow-up of a of minimum three years. No successive surgical treatments were required at any time. No complications related to the bioglass were detected [71]. The authors of the research did not search for signs of local and systemic inflammation.

Further, 182 patients with osteonecrosis of the femoral head were randomly divided into four groups: implanted autogenous fibula graft (FFG), free vascularized fibular graft (FVFG), fresh bone marrow was collected and mixed with an autologous iliac bone graft (ABG) and β-TCP bioceramics particles and rod were implanted into the canal [72]. The results showed no significant difference in baseline data among the four groups. All patients were followed up for 42 to 48 months. Three hips collapsed on the femoral head in the FFG group, two in the FVFG group, two in the ABG group and three in the β-TCPG group [72]. Thus, the results of the implant are comparable to the gold standard for implantation by allograft. The research did not examine markers of local and systemic inflammation.

In another randomized clinical trial, forty patients (51 hips) with avascular necrosis of the femoral head were randomly divided into two groups [73]: group A received core decompression, autologous bone-marrow-aspirated buffy coat and angioconductive bioceramic rod grafting; group B received treatment of core decompression with β-TCP granules and angioconductive bioceramic rod grafting. The clinical failure rate was 4.5% (1/22) and 17.2% (5/29) in groups A and B, respectively. Kaplan–Meier analysis did not show obvious statistical differences in survival (*p*  =  0.203) but suggested a trend that survivorship of the femoral head in group A is higher than that in group B [73]. In the study, immune responses were not examined.

Thus, various organs, localized both internally and at the surface of ceramic biomaterials, have shown improved tissue regeneration in animal models. Promising results of pre-clinically obtained animal models lead researchers to the next step of clinical studies on patients. Additionally, the results of studies of various bioceramics on humans are not unambiguous and leave a wide scope for further research. However, there is an urgent need to understand how local immunity, defined predominantly by tissue-resident macrophages or infiltrated monocytes, will interact with these newly developed ceramic material delivery systems. Currently available methods in quantitative immune histology, confocal microscopy and spatial transcriptomics have to be applied in combination with ex vivo modelling to decipher the mechanism of interaction of ceramic biomaterials with primary human immune cells.

## 3. Benefits and Problems of Implant Materials and Composition (Mechanical)

The mechanical properties of an implant define its application in different medical fields for different purposes. To assess the mechanical properties of materials used in implantology, the following parameters are utilized:

- Compressive strength or compressive strength is the ability of a material or structure to resist loads tending to reduce the size.
- Young’s Modulus (GPa) is the ability of the material to resist tension and compression under elastic deformation.
- Poison’s ratio is a measurement of the deformation (expansion or contraction) of the material in the directions perpendicular to the specific direction of loading.
- Flexural strength (MPa) is the ability of a material to resist bending failure.
- Tensile strength (MPa) denotes the maximum mechanical tensile stress.
- Corrosion is spontaneous destruction of metals and alloys as a result of chemical and/or physical interaction with the environment.
- The wear rate is the change in size, shape, mass or surface condition of a product or tool due to the failure of the product’s surface layer under friction [74,75,76].

The microarchitecture as well as composition of the implant affect its mechanical properties. A porous zirconia scaffold has a lower Young’s modulus and compressive strength than a monolithic zirconia implant. However, in order to compare porous implants made of different materials, it is necessary to compare the porosity and particle size values of the implants. For some types of implant materials, no measurement of mechanical parameters was performed in the form of scaffolds, so it is impossible to make a comparative assessment of them. Aluminum and zirconia ceramics show higher compressive, flexural, tensile and Young’s modulus values compared to those of HA and β- TCP ceramics, bioglass and natural bone (Table 2), but specific values significantly depend on the individual studies. Thus, compression strength of bioglass ranges from 1.7 to 140 MPa and the tensile strength of HA ceramics ranges from 38 to 300 MPa (for summary references, see Table 2).

Material particles resulting from metallic wear (macro-, micro- and nanoparticles) are an important parameter to consider in implant manufacturing for all types of applications, including arthroplasty. Released debris particles can activate undesired immune reactions, but we found no studies analyzing this parameter for most metal ceramic materials. Data on the resistance to wear of metal implants is variable, and metal implants are also susceptible to corrosion, which also leads to the formation of immunogenic particles. Metal wear occurs due to displacement of surface material and detachment of debris particles, metal corrosion is the destruction of metals as a result of an oxidation reduction process [83,84,85,86,87,88].

The susceptibility of metal implants to corrosion is an essential limitation that argues towards development of the materials, including new ceramic variants, that would improve implant stability and biological integration by reducing the corrosion rate. Major directions for such improvements include creating new corrosion-resistant materials and coating metals with corrosion-reducing particles and surface modification [88,89,90,91,92,93,94,95]. When comparing the performance of monolithic (non-porous) aluminum ceramics, the zirconium (Zr) scaffolds are the closest to the performance of trabecular bone. Porous bioglass implants are closest to both trabecular and cortical bone (Table 2).

Young’s Modulus (GPa) is the highest in non-porous aluminum ceramics, the closest to native trabecular bone is HA ceramics and bioglass is the closest to cortical bone (Table 2). The Poisson’s ratio of most ceramic materials is close to the value of the corresponding coefficient of native bone with the exception of aluminum ceramics (Table 2). The highest value of Flexural strength is for Zr ceramic, the closest to the trabecular bone is non-porous HA implant and to the cortical bone is non-porous β-TCP ceramic.

It is impossible to choose one material with the highest mechanical properties or the closest mechanical properties to native bone. When choosing an implant material, we should define the properties needed for the implant in certain medical fields and in certain pathologies. For example, if an implant is planned in segments with high axial load, use of HA ceramic-scaffold-type implants is currently impossible, but bulk-type titanium implants can be used.

The most-required mechanical properties that are currently being developed in leading laboratories include compressive strength, bending strength, tensile strength, durability and fatigue resistance to meet mechanical stress [3,87].

## 4. Immune Response to Bioceramic Implants

Any material placed in the living body will be recognized by the immune system and can induce an immune response. Despite their high biocompatibility, ceramic implants also cause a variety of local and systemic immune responses, which may be easily detectable by overt clinical symptoms or may proceed as low-grade, latent inflammation. All types of ceramic implants can potentially cause an inflammatory response in the body (summarized in Table 3; see also specific reference). Currently available methodology also provides high sensitivity to detect low-grade inflammation; however, it does not provide sufficient precision to distinguish between specifics for each implant type’s detrimental effects on the local and system innate immunity. Such specific reactions are defined, first of all, by the direct material, surface structure and shape recognition by the resident tissue macrophages [96].

Implant placement is accompanied by an immediate and delayed inflammatory reaction, with monocytes/macrophages and neutrophils participating in inflammatory responses. The immediate/hot innate immune response within 4–6 h is characterized by precipitation of blood proteins (implant opsonization), platelet aggregation, complement activation and, in some cases, involvement of mast cells, NK, T cells and B cells [126]. The delayed immune response can be caused by overactivation of innate immunity (first macrophages) and can also involve B and T lymphocytes [127]. Such unbalanced and delayed inflammation is characterized by an unbalanced production of pro-inflammatory factors (TNFα, IFN-γ, IL-2, IL6, IL8, IL12) and anti-inflammatory/pro-healing/pro-fibrotic factors (IL-4/IL13, IL-10, TGF-β, GDF15) [128,129].

Macrophages are key regulatory cells at all stages of tissue/implant interaction. In the ideal case, macrophages should switch off the inflammatory reactions and ultimately induce the healing program in the tissue [130]. However, due to their inability to destroy the implants, macrophages stay permanently in the status of “frustrated phagocytosis”, which can be accompanied by the undesired release of cytokines and enzymes. The spectrum of the detrimental macrophage reactions will depend on the implant characteristics as well as the status of the immune system (both local and systemic) of the patient. For example, implant integration in diabetic patients is significantly compromised by pro-inflammatory programming of innate immunity by metabolic factors (hyperglycemia, dyslipidemia) [131,132,133,134,135].

In the case of ceramic implants, all types of inflammatory reactions have been reported (for summary and references, see Table 3). Several studies on different animal models demonstrate that ceramic materials induce an acute inflammatory response on the first few days after implantation, but later, the inflammatory response decreases rapidly [79,136,137,138].

Sterilized bioceramic discs were implanted subcutaneously into the interscapular area of 32 rats; then, histological reactions were quantified by light microscopy and observed from 1 day to 300 days after implantation [138]. The control group underwent surgery without inserting the disc and underwent similar histological studies. The results showed that various numbers of inflammatory cells, including neutrophils, macrophages, lymphocytes, giant foreign body cells and fibroblasts and fibrocytes, accumulated around the bioimplants. One day after implantation, peri-implant tissues were dominated by macrophages (69.8%), followed by neutrophils (19.7%) and lymphocytes (8.8%); no proliferation of fibroblasts and fibrocytes was observed. Three days after implantation, there was a decrease in the neutrophil level to almost zero (0.49%), and macrophages and lymphocytes decreased to 53.3% and 4.4%, respectively. Seven days after implantation, fibroblasts and fibrocytes were detected, macrophages and lymphocyte levels continued to decrease and no neutrophils were observed. The results 14 days after implantation showed a persistently low level of macrophages and lymphocytes, the level of fibroblasts began to decrease and only the level of fibrocytes continued to increase [138].

In the control group, macrophages were present 1 day after surgery, but they completely disappeared 7 days after surgery. Neutrophils were only visible on day 1 postoperatively. Fibroblasts and fibrocytes gradually increased after 3 days [138].

In the experimental group, from 30 to 300 days after implantation, there was a steady decline in macrophages and lymphocytes to very low levels, and the capsules of fibrous tissue around the implants were maturing. At later stages, implants were surrounded by mature fibrous capsules consisting of collagen with fibrocytes and fibroblasts [138].

However, the clear beneficial features for ceramic implants are the low frequency of bacterial contamination (in most cases, implant-induced inflammation is sterile) and reduced intensity of the acute inflammatory phase [79,139]. Acute periprosthetic inflammatory reaction is more typical for “metal-on-metal” implants than for ceramic implants [140,141]. It is characterized by soft tissue necrosis, infiltration of T-cell and B-cell lymphocytes, as well as accumulation of plasma cells and macrophages and abnormal articular fluid [140,141].

Intensive chronic inflammation is more typical for alumina and zirconia implants, while low-grade inflammation can be caused by all ceramic implants, indicating that further significant improvement is needed to overcome innate immune reactions on a long-term basis. Aluminum oxide particles showed a moderate nonspecific granulomatous reaction of the synovial membrane in the knees of rats in vivo, which increased the production of IL-1ß and MCP-1 (CCL2) when interacting with macrophages [142]. Chronic low-grade local inflammation was detected in 44 patients recruited at the University of Zurich, Switzerland, with a single missing tooth replaced by a zirconium implant between 2011 and 2013 within 6 months after the installation of dental zirconium implants [143]. Patients (22 men; 22 women), average age 49.1 years (range 21.3–81.4 years), had no moderate/severe systemic disease or bruxism. All patients had good oral hygiene, including smokers and nonsmokers. Analysis of samples of peri-implant tissues taken during histological examination revealed a large number of fibroblasts and fibrocytes (part of granulation tissue), as well as inflammatory cells (monocytes, lymphocytes and macrophages) [143]. This study did not address specific types of macrophage activation, but only the total number evaluated by optical microscopy for descriptive histology with semiquantitative analysis [143].

The application of at least three major ceramic implant materials, alumina, zirconia and titanium, can also be accompanied by tissue destruction [111,112,113]. These failures are classified as aseptic or septic. They are associated with the presence of implant wear particles. Aseptic loosening is the result of chronic inflammation caused by the activation of immune cells in contact with implant wear particles. Septic loosening is determined by the presence of chronic infection at the implant site. However, studies show the assessment of the cause of inflammation can be misleading. The study shows the effect of wear debris on the formation and survival of a biofilm [111]. Routine microbiological diagnosis can misdiagnose aseptic loosening. The interaction of implant wear particles with macrophages and neutrophils impairs the ability of the innate immune system to remove bacteria. In addition to disrupting the innate immune system, wear particles destroy dendritic cells and T lymphocytes of the adaptive immune response [111]. This aggravates both aseptic and septic loosening, causing chronic inflammation and greater osteolytic resorption. It can be assumed that tissue destruction can be caused by more than simply inflammatory activity of macrophages in a state of “frustrated phagocytosis”, and it has to be investigated in the future for the mechanism and biomarkers.

Despite the fact that inflammation associated with ceramic implants is most often sterile, isolated studies also report cases of inflammation caused by bacterial contamination.

Postoperative follow-up of 1549 patients (991 men, 558 women) with porous HA implants for cranioplasty showed infectious complications in 33 cases [99]. The patients were 7–87 years old, with an average age of 32 years. Implants were placed predominantly in Europe (Germany, France, Italy); eight were placed in the Middle East, six in Africa, two in Canada, one in Central America, one in Oceania and one in South America. Infectious complication was more common in patients with head trauma (27 patients). The development of an infectious complication occurred at different times: less than 6 months in 22 patients, within 6–12 months in 2 patients and after 1 year in 9 patients. In 18 cases of infection, the implant was reinstalled after treatment; the study did not indicate whether the infectious agent was determined [99].

Inflammatory reactions that are specific for ceramic implants are currently insufficiently investigated, mostly due to the superficial methods to assess inflammatory reactions. For example, inflammatory status was analyzed in peri-implant tissue obtained from patients during the planned removal of the implant from yttrium-stabilized zirconium oxide (Y-TZP), aluminum-oxide-reinforced zirconium oxide (ATZ), aluminum-oxide-reinforced zirconium oxide (ZTA) or titanium [144]. The presence of macrophages, B lymphocytes, T lymphocytes and plasma cells (antibody-secreting cells) was detected in all samples by quantitative histological analysis using ImageJ software with CD3 1:200 (T lymphocyte), CD20 1:400 (B lymphocyte), CD138 1:50 (plasma cell, clone MI15), CD68 1:200 (macrophage, clone PG-M1) biomarkers. The authors did not find significant differences in the number of inflammatory cells around ceramic or titanium implants [144]. However, this study was limited to very general cell-type biomarkers, and specific biomarkers for macrophage subpopulations that are highly diverse in their phenotypes and functions have not been assessed. The number of patients examined was also limiting for making quantitative conclusions [144].

In an experimental animal model, ceramic materials were compared to metal and polymeric particles with regard to local inflammatory complications during histological examination of peri-implant tissues. All types of materials were placed in the right femur of the rat [145]. This study compared particles made out of ceramics (Al2O3, range size 0.2–6.3 µm), chrome cobalt (Co–Cr—0.2–5.6 µm), titanium alloy (Ti–6Al–4V—0.2–3.8 µm) and ultrahigh-molecular-weight polyethylene (UHMWPE −0.2–9.5 µm). Assessment of inflammation was performed 16 weeks after implantation. As a quantitative parameter for the level of inflammation, the thickness of inflamed tissues was determined histologically using a computerized image analysis system. The analysis of the inflammatory tissue thickness between the animal groups with different types of particles showed that both UHMWPE and ceramic particles significantly led to the formation of inflammatory periprosthetic tissue [145].

There are reports of type IV allergic reactions to all ceramics, with the exception of aluminum and bioglass (Table 3). A type IV allergic reaction is a delayed reaction mediated by a cellular response [146]. It is mediated by T cells that provoke an inflammatory response against various antigens. In some situations, other cells, such as monocytes, eosinophils and neutrophils, may also be involved. After exposure to the antigen, an initial local immune and inflammatory response occurs, which attracts leukocytes. The antigen absorbed by macrophages and monocytes is transmitted to T cells, which then become sensitized and activated. These cells then release cytokines and chemokines, which can cause tissue damage and lead to disease. Type IV reactions are divided into IVa, IVb, IVc and IVd, depending on the type of T cells (CD4 T-helper type 1 and type 2) and the cytokines/chemokines produced [146]. Conflicting data were published for the allergic reactions to β-TCP [123,124,147]. Depending on the manufacturer of the bioceramic material, different researchers reported/did not report allergic reactions [123,124]. A delayed-type allergic reaction was detected for calcium-phosphate ceramics in C57BL/6 mice that were observed 2 and 4 weeks after ceramics administration [123]. Antigenic specificity was demonstrated between calcium-phosphate ceramics and foetal bovine serum in cross-over. The adverse reaction was manifested as swelling of the paw pads. Because of the reaction time, the authors of the study assumed a delayed-type allergic reaction [123]. In another study, the antigenicity of β-TCP ceramics was studied using delayed skin reactions in guinea pigs. Skin reactions appeared 13 days after immunization by intradermal injection of ceramics into the dorsal sides of guinea pigs. After 24 and 48 h, these reactions were evaluated by measuring the diameter of the erythema, the degree of haemorrhage and its induction. However, no detailed study of the reaction mechanism has been performed [124].

Positive paw pad reactions to β-TCP ceramics were shown in C57BL/10 (H-2b) and C57BL/10 X BR (H-2k) but were not observed in C57BL/10 X D2 (H-2d). However, the mechanism of the allergic reaction has not been investigated [124].

Despite the absence of clinical symptoms of systemic inflammation, when ceramic implants are placed, we found a study claiming the presence of markers of systemic inflammation [114]. The biomarkers used characteristics for all types of traumatic injury, and their increase in circulation can be simply caused by surgical intervention. However, an interesting observation was made for the 105 patients (123 affected teeth), age range 18–65 years, with good oral hygiene habits and high treatment compliance within 12 months from implant placement [114]. A statistically significant increase in the levels of YKL-40, resistin, aspartate aminotransferase (AST) and alkaline phosphatase concentrations was detected in the gingival sulcus fluid [114]. The protein levels for all these biomarkers were statistically significantly lower in the zirconia crown (ZC) group compared to the cobalt–chromium metal (MCC) [114].

Among the markers tested in this study, YKL-40 is of particular interest. YKL-40 (CHI3L1), or human cartilage glycoprotein 39 (HC gp-39), belongs to the family of chitinase-like proteins that biochemically possess lectin properties but lack the hydrolytic activity characteristic for true chitinases [148]. YKL40 can be secreted in high amounts by tissue macrophages and is induced by IFN gamma; however, its biological activities can be rather categorized as pro-healing (stimulation of cell proliferation, stimulation of angiogenesis [149,150]). Elevated levels of circulating YKL-40 were found in patients with cancer, cardio-metabolic disorders, neurodegenerative disorders, and autoimmune disorders, which are major pathologies that progress in the background of chronic low-grade inflammation in various tissues [151,152,153,154,155,156]. Based on its mode or expression regulation and biological effects, YKL-40 can be considered as a systemic biomarker for local, frequently low-grade mixed-type inflammation, where pro-inflammatory and healing processes interfere with each other. Therefore, elevated levels of YKL-40 in the circulation of patients with ceramic implants indicate that the immune reaction to the implant is not silent and develops in a detrimental inflammatory direction.

## 5. Prospects for Affecting Immune Response through Implant Modification

**Figure 2:**
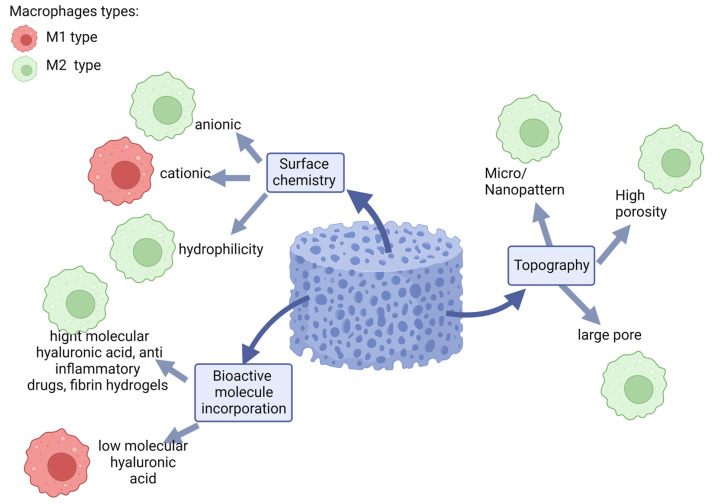
Effect of biomaterial modifications on macrophage polarization.

Several approaches are being elaborated and show the possibility to influence the activation of macrophages towards a specific functional phenotype (also named macrophage polarization) by changing the structure (porosity, pore size, etc.), surface chemistry and adding different agents to the implant (Figure 2). We can define three main directions for modeling the implant to regulate the macrophage response: changing the surface chemistry of the implant, changing the structure of the implant and adding bioactive molecules to the implant. Thus, the hydrophilicity and anionic charge of the surface contribute to the polarization of predominantly M2 macrophages. The large pore size, high porosity and nanopattern of the implant also promote the proliferation of M2 macrophages.

Physicochemical properties of biomaterials and the topography of their surface affect the microenvironment of implants, thereby affecting the intensity and direction of the anti- or pro-inflammatory reaction [157,158]. Modifications to the implant surface, both physical and chemical, are utilized to control the inflammatory response and tissue regeneration, primarily by directing the polarization of macrophages [129,159,160,161,162,163,164,165] (Table 4).

Brodbeck et al. found correlation between implant surface charge and proliferation of different types of macrophages [180]. Human monocytes in vitro were cultured on a surface modified to hydrophobic, hydrophilic, anionic and cationic surface properties. Semiquantitative RT-PCR analysis on days 3, 7 and 10 of cell culture showed that Interleukin-10 (IL-10) expression was significantly increased in cells adherent to the hydrophilic and anionic surface, but significantly decreased in monocytes/macrophages adhering to the cationic surface. Interleukin-8 (IL-8) expression was significantly decreased in cells adhering to hydrophilic and anionic surfaces [180]. Bioceramic implants also have a surface charge. The surface chemistry of HAs is mainly related to the rate of SO_4_^2−^ substitution at the PO_4_^2−^ site [181]. A nanoscale element with a reduced negative surface potential affects protein adsorption through weak repulsive or attractive forces. Adsorption studies of bovine serum albumin (BSA) and lysozyme (LSZ) confirmed an increased affinity for active binding sites on the HA surface [181]. Thus, the effect of surface charge in the direction of macrophage polarization may also be true for ceramic implants, which needs further investigation.

Research links the hydrophilicity of the implant material to the direction of macrophage polarization, affecting protein adsorption [158,166,167]. Titanium implants with micro- and hydrophilic roughness (SLA) or hydrophilic-modified rough surfaces (modSLA) were examined in vitro by rodent and human macrophages [182]. Non-adherent cells were harvested and cultured.

As a result of incubation, they were transformed into M1 or M2 macrophages. The M1 and M2 phenotype was confirmed by cytokine secretion analysis of IL-1 and IL-10, respectively, using ELISA. However, a significant titanium surface effect was observed, in which significantly lower levels of these cytokines were secreted by M1 and M2 cells cultured on the modSLA surface.

IL-10 levels were significantly elevated when M2 cells were cultured on the modSLA surface compared to the same cells on the SLA surface [182]. We did not find studies examining the effect of different ceramic hydrophilicity on macrophage proliferation, but HA ceramics are used to increase the hydrophilicity of titanium implants and, therefore, have the potential to influence macrophage polarization [182]

The nanostructure and rough surface of the material has a positive effect on the anti-inflammatory development of the immune response, regardless of the material of the placed implant [158,166,167,168,169,170,171,172]. On the rough surface of the implants (including ceramics), macrophages have a tendency to develop the M1 phenotype, while smoother surfaces guide macrophage towards M2 polarization [183].

HA bioceramic discs with different roughness and grain sizes were implanted into rectus femoris muscle BALB/C mice. Seven days after the surgery, five samples were harvested from each group [184]. The concentrations of inflammation-related cytokines secreted from the macrophages, including TNF-α, IL-1β, IL-10, MIP-1α, MIP-1β and MCP-1, were determined in the collected supernatants, following the manufacturer’s instructions for the commercial ELISA kits. The nano-structured highest roughness and smallest grain-size group had the highest secretion of anti-inflammatory cytokine IL-10 on day 3 and the lowest secretion of pro-inflammatory cytokine TNF-α on days 3 and 5 (*p* < 0.05). The immunomodulatory effect of the micro/nano-hierarchical structures was studied by the flow cytometry detection of polarized RAW 264.7 surface markers CCR7 and CD206. The ratio of CCR7-labeled M1 phenotype to CD206-labeled M2 phenotype was also calculated. The nano-structured highest-roughness group induced the lowest proportion of CCR7-positive M1 macrophages and the highest proportion of CD206-positive M2 macrophages (*p* < 0.01) [184].

Studies have shown that pore size and high porosity also affect the polarization of macrophages, regardless of the implant material [128,158,173]. Thus, 3D-printed polycaprolactone/polyethylene glycol/ HA scaffolds were, in vitro, tested on RAW 264.7 cells. The cells adhering to the scaffolds were observed using SEM [173]. Further, implants with different porous sizes (including 209.9 ± 77.1 μm (P200), 385.5 ± 28.6 μm (P400) and 582.1 ± 27.2 μm) were placed on mandibular bone defects in 32 SD rats (four rats per group). The left mandible implantation area of the rat was shaved and disinfected. Rats were sacrificed at 4 and 8 weeks after implantation, and the mandibular specimens were used for micro-computed tomography (micro-CT) examination and immunofluorescence staining. In this study, decreased amounts of inflammatory cells were recruited in the large pore group compared to in the small pore groups. Neutrophils and macrophages were observed surrounding the surface of the scaffold and in pores [173]. On day 3, a significant difference in the total number of inflammatory cells was not observed among the groups. On days 7 and 28, in the largest pore size group, the number of inflammatory cells was significantly lower than that in the other groups. The M2/M1 ratio of the largest pore size scaffold group was obviously higher than that of the other groups on day 7 and day 28, and the difference was more obvious on day 28 [173].

The hydrophilicity of different material-type implants also affects the polarization of macrophages, and hydrophilic implants enhanced the expression of M2 [182,185,186].

The application of hyaluronic acid to the implant surface can have a different effect on inflammation. High-molecular hyaluronic acid has anti-inflammatory and immunosuppressive effects; low-molecular hyaluronic acid can act as a powerful pro-inflammatory molecule [127]. Hyaluronic acid has an anti-inflammatory effect by decreasing TNF-α and reducing the infiltration of activated neutrophils (in vivo study in rats), suppressing MMPs and ADAMTS, interacting with CD44, COX-2 and PGE2 (in vitro study) [187].

Adding bioactive molecules with known properties regarding macrophage proliferation to the implant seems to be an obvious way to modify the implant [136,178,188,189]. However, the example of hyaluronic acid proves that the same substance can have different effects on the properties of the implant. Hyaluronic acid binds to ECM molecules and cell surface receptors, thereby regulating cell behavior through the control of the tissue macro- and microenvironment. Hyaluronic acid can bind to three major classes of cell surface receptors: CD44 (membrane glycoprotein), receptor for hyaluronate-mediated motility (RHAMM) and intercellular adhesion molecule 1 (ICAM-1). CD44 interacts with a number of other ligands, including osteopontin, collagens and matrix metalloproteinases (MMPs). High- and low-molecular-weight HAs have different molecular and cellular mechanisms and a variety of biological effects through interaction with CD44 receptors, which explains the opposite effects of hyaluronic acid as an implant surface modifier. In addition to these receptors, two other receptors have been identified for HA binding: lymphatic endothelial hyaluronan receptor (LYVE-1) and hyaluronic acid receptor for endocytosis (HARE), also known as Stabilin-2 [187,190]. The use of fibrin hydrogels on the surface of HA ceramic implants has a strong stimulating effect on the recruitment of anti-inflammatory macrophages M2 [136]

Thus, there are many prospects for implant modification, but the large number of variants and their combinations require experimental testing of the effect on the immune response. Perhaps the use of mathematical models will reduce the number of perspective variants for research.

## 6. Mathematical Models for Data Integration

The most common mathematical models consider the processes of bone tissue remodeling and implant osseointegration. They represent differential equations, in which the unknown functions are populations of various cells (mesenchymal stem cells, osteoblasts, fibroblasts, chondrocytes, osteoclasts, etc.), concentrations of various biochemical factors (osteogenic, chondrogenic, vascular, etc.) as well as tissue density (fibrous, cartilage, bone, etc.) near the fracture area or the implant [191,192,193,194,195]. In most scientific works, the results of numerical solutions of mathematical model equations and computer modeling show good qualitative and quantitative coincidence with the results of laboratory and clinical studies. For example, the mathematical model describes the dynamics of osteoblasts and osteoclasts during bone remodeling and takes into account the effect of various drugs (teriparatide, denosumab and romosozumab), demonstrating good agreement between the results of numerical simulation and clinical data for patients with osteoporosis [196]. Another work used mathematical modeling to predict the effect of Wnt-10b protein administration on bone tissue metabolism and to validate the results with data from laboratory experiments on mice [197].

Less work is devoted to the mathematical modeling of the complex of immune and inflammatory reactions that start in the organism immediately after the implant placement. Using a mathematical model, we were able to qualitatively describe some features of the body’s reaction to a foreign body and confirm the important role of macrophages in this process [198]. Two-dimensional numerical modeling allows one to obtain quantitative estimates of collagen deposition on bioimplants. Another mathematical model is devoted to a detailed description of the behavior of macrophages, fibroblasts and their interaction during fibrous tissue formation [199]. The authors note that the model they propose does not include all types of reactions arising in the immune response and, therefore, can be considered only as an approximation. Various mathematical models of macrophage and cytokine dynamics during bone healing and after implant placement have been proposed in recent works [200,201]. Thus, we can conclude that mathematical models that describe the complex dynamics of the body’s immune response to implants made of different materials are currently underdeveloped.

## 7. Conclusions

In summary, ceramic implants provide one of the best therapeutic solutions for multiple clinical applications due to their extremely high compatibility with the regenerative processes. However, inflammatory and tissue-destructive processes cause complications and restrict the probability of application of these implant materials.

In contrast to other types of materials (metals or polymers), insufficient experimental attention was given to the examination of specific immune reactions to ceramic materials, which, in hand, significantly limits further research and the development of improved ceramic implants with life-long integration potential. Recent advances in the application of the cellular ex vivo test systems, in particular the model systems based on human primary cells, as well as significant progress in the analytics of immune cell activation (single-cell transcriptome, metabolome and epigenome analysis) offer the possibility of addressing the specificity of immune reactions on ceramic effects on the most precise level. The data that will be obtained will be complex, and their integration needs the involvement of complex mathematic modelling.

Mathematical models that describe the complex dynamics of the body’s immune response to implants made of different materials also have to be upgraded and further developed, since currently available models consider extremely limited numbers of immune parameters. Development of new mathematical models that would adequately describe the specific immune responses of the body to bioceramic implants, taking into consideration the cross-talk between physical characteristics, surface architectures, chemical modifications of the ceramic material and activation of multiple immune cell types that interact with the biomaterials by direct contact and by the release of cytokines, growth factors, chitinase-like proteins, ECM structural components and ECM-modifying enzymes, is an urgent task in the field of biomedical mathematical modelling.

## Figures and Tables

**Table 1:** Types for ceramic materials for implants.

	Type of Material	Medical Field	Pathology	Limitations of Material	Complications	Delayed Inflammation	Ref
Non-absorbable	alumina (Al_2_O_3_)	Orthopedic implants	bone damage, arthrosis	non-osteointegrative	to develop a nonadherent fibrous membrane at the interface, encapsulation aseptic loosening	yes	[3,37,38]
	zirconia ceramic	Orthopedic implants, dental implants	bone damage, arthrosis, teeth issues	low thermal conductivity, non-osteointegrative	bone resorption and increased fracture risk aseptic loosening	yes	[37]
	Titanium ceramic	Orthopedic implants dental implants	bone damage, bone cancer	poor mechanicaquality, mismatch of mechanicaproperties	allergic reaction aseptic loosening	yes	[39,40,41]
Biodegradable /Bioactive	HA	Orthopedic implants, skin implants, respiratory implants, drug delivery system	bone damage, hepatocellular carcinoma, lung cancer, bone cancer, breast cancer	Fragility	uncontrolled bone resorption	yes	[3,31,38,42,43,44]
	β-tricalcium phosphate	Orthopedic implants, skin implants, dental implants, drug delivery system	bone damage, osteoporosis bone cancer, dental issues	poor fatigue resistance and brittleness	uncontrolled bone resorption	yes	[30,43,45,46]
	Bioglass	Orthopedic implants, skin implants, respiratory implants, cardiovascular implants, Neurological implants, drug delivery system	spinal fusion, Cutaneous wounds, osteoporosis, Bone cancer, myocardial necrosis, chronic obstructive pulmonary disease, peripheral nerve injuries, Gastric ulcers	slow degradation, fragility	Causes ankylosis and decreased fracture resistance	Yes	[11,30,37,41,44,47,48,49]

**Table 2:** Mechanical properties of ceramic implants in comparison with native bone and metal implants. “+” has the property; “-” has no property, “?” indicates that no information has been found.

Material	Compressive Strength (MPa)		Young’s Modulus (GPa)		Poison’s Ratio	Flexural Strength (MPa)		Tensile Strength (MPa)	Corrosion	Average Wear Rate of the Placed Implant	Ref
	Bulk	Scaffold	Bulk	Scaffold		Bulk	Scaffold				
Alumina Ceramic	4500	-	300–400	-	0.21–0.22	379	106.2	350	-	1 μm/year	[3,74,77,78,79]
zirconia ceramic	2500	0.6–2.04	210	0.78	0.30	1100	-	650	-	?	[3,24]
Titanium ceramic	?	-	53	-	0.27–0.32	?	-	665	+	?	[3,24]
HA Ceramic	300–900	3.44–5.98	0.17–0.26	0.17–0.26	0.27	9	-	38–300	-	?	[3,24,79,80,81]
β–TCP Ceramic	292	21.3	80–162	-	0.22–0.29	147	-	-	-	?	[3,79]
bioglass	500	1.7–140	35	13.2	0.26–0.39	70	11	42	-	?	[3,79]
Trabecular bone	0.1–50	N/A	0.05–0.5	N/A	0.25	10–20	N/A	60–160	N/A	N/A	[3,82]
Cortical bone	30–200	N/A	7–30	N/A	0.3	50–150	N/A	50	N/A	N/A	[3,82]
Stainless steel	170–310	-	200–210	-	0.29–0.3	170–310	-	480–620	+	?	[3,74,75,76]
Titanium based alloys	130	-	102.7–104.1	-	0.35	172–240	-	240–550	+	?	[3,74,75]

**Table 3:** Type of immune response on ceramic implants. “+” has the type of reaction “-” has no the type of reaction.

Types of Inflammation	Alumina (Al_2_O_3_)	Zirconia Ceramic	Titanium Ceramic	HA	β-Tricalcium Phosphate	Bioglass + (Type IV)	Ref
Sterile or bacterial (what kind of)	Mostly sterile	Mostly sterile	Mostly sterile	Mostly Sterile Neisseria Meningitidis (one report available)	Sterile	Sterile	[97,98,99,100,101]
Chronic intensive	-	-	+	No reports	No reports	No reports	[102,103,104,105,106,107,108,109,110]
Chronic low grade	+	+	+	+	+	+	[100,109,111,112,113,114,115,116,117,118,119,120,121,122]
Allergic	No reports	+ (type IV)	+ (type IV)	No reports	+ (type IV)	No reports	[121,123,124,125]
Tissue destruction without clear inflammation	+	+	+	No reports	No reports	No reports	[111,112,113]

**Table 4:** Biomaterial modification for modulating immune responses. ”+” applicable, “NA” not analyzed.

Engineering Parameters	Modifications	Outcome	Applicable for Ceramics	
Surface chemistry	Surface charge (anionic)	↑IL-10, ↓IL-8	+	[158]
	Surface charge (cationic)	↓IL-10, ↓IL- 1RA	+	
	Hydrophilicity	↑IL-4, ↑IL-10, ↑TGF-β, ↑BMP2 ↓TNF-α, ↓IL-1β, ↓IL-6	+	[158,166,167]
Topography	Micro/ Nanopattern	↑IL-10, ↑IL-4, ↑IL-13, ↓TNF-α, ↓IFN-g	+	[158,166,167,168,169,170,171,172]
	High porosity	↑Arginase	+	[158]
	Large pore	↑Arginase, ↓iNOS, ↓IL-1R1	+	[128,158,173]
	Roughness	↑IL-4, ↑IL-10, ↑IL-11, ↑IL-13	+	[166,167,172,174,175,176] [177]
Bioactive molecule incorporation	Proteins	↑ (BMP-2), ↓iNOS, ↓IL-6, ↓IL-1β	+	[158]
	Nucleic acids	↓ (MALAT1),↑IDO	+	[158]
	Anti inflammatory drugs	↑IL-10, ↓IL-1β	+	[158]
	Cytokines	(IL-4) ↓TNF-α	+	[158]
	Cytokines (OSM)	↑STAT-3, ↑ALP		[158]
	highly sulfated hyaluronan (HA)	↓IL-6-, ↓IFN-g↓, MCP-1 ↑IL-10	+	[127]
	Hyaluronic acid (HA)	↓TNF-α, ↓IL-1, ↓IL-6.	+	[178]
	fibrin hydrogels	↓TNF-α ↑ IL-10	+	[136]
	grafted unsaturated polyurethane films	↑IL-10, ↑TGF-β	N/A	[179]

## Abbreviations

HA, hydroxyapatite; β-TCP, beta-phase tricalcium phosphate; IL-1RA, interleukin-1 receptor antagonist; TGF-β, transforming growth factor beta; BMP2, Bone morphogenetic protein-2; TNF-α, Tumor necrosis factor alpha; iNOS, inducible nitric oxide synthase; IDO, indoleamine 2,3-dioxygenase; IFN-g Interferon gamma, MCP-1-Monocyte chemoattractant protein-1; OSM, oncostatin M; ALP, alkalinephosphatase; PLA, poly lactic acid.
